# Clinicopathologic features of incidental prostatic adenocarcinoma in radical cystoprostatectomy specimens

**DOI:** 10.1186/1477-7819-9-81

**Published:** 2011-07-20

**Authors:** Berna Aytac, Hakan Vuruskan

**Affiliations:** 1 Department of Surgical Pathology, Uludag University Medical Faculty, Bursa, Turkey; 2 Department of Urology, Uludag University Medical Faculty, Bursa, Turkey

**Keywords:** Bladder cancer, cystoprostatectomy, incidental, prostate cancer

## Abstract

**Background:**

The aim of this study is to review all features of incidentally discovered prostate adenocarcinoma in patients undergoing radical cystoprostatectomy for bladder cancer.

**Methods:**

The medical charts of 300 male patients who underwent radical cystoprostatectomy for bladder cancer between 1997 and 2005 were retrospectively reviewed. The mean age of the patients was 62 (range 51-75) years.

**Results:**

Prostate adenocarcinoma was present in 60 (20%) of 300 specimens. All were acinar adenocarcinoma. Of these, 40 (66.7%) were located in peripheral zone, 20 (33.3%) had pT2a tumor, 12 (20%) had pT2b tumor, 22(36.7%) had pT2c and, 6 (10%) had pT3a tumor. Gleason score was 6 or less in 48 (80%) patients. Surgical margins were negative in 54 (90%) patients, and tumor volume was less than 0.5 cc in 23 (38.3%) patients. Of the 60 incidentally detected cases of prostate adenocarcinoma 40 (66.7%) were considered clinically significant.

**Conclusion:**

Incidentally detected prostate adenocarcinoma is frequently observed in radical cystoprostatectomy specimens. The majority are clinically significant.

## Background

Prostate adenocarcinoma (PCa) is the most common visceral malignancy in the male population and the second leading cause of death in men [1]. It can be found incidentally when the prostate is removed during radical cystoprostatectomy (RCP) for bladder cancer and latently at autopsy or clinically diagnosed by physical examination, laboratory tests, and symptoms [2,3]. In autopsy series incidental prostate cancer is found in 30% of men in their fifth decade and that rate increases to as high as 90% in men aged older than 90 years [4]. The frequency of PCa incidentally discovered in RCP specimens is extremely variable, ranging from 10% to nearly 60% [1,3,5]. These tumors are typically small, well- or moderately well-differentiated, localized entirely within the gland, and most being regarded as clinically insignificant [3,6].

Our aim was to review features of incidentally discovered prostate adenocarcinoma in patients with bladder cancer with regard to their incidence, pathologic characteristics and clinical significance.

## Methods

We reviewed the medical charts of 300 men who diagnosed muscle-invasive bladder urothelial carcinoma and no history or clinical evidence of PCa before surgery in 1997-2005. Of these, 60 patients who had concomitant PCa were included in our study. Physical examinations, laboratory studies, chest radiographies and abdomino-pelvic computed tomography were performed in all patients. The clinical records were obtained at the time of admission, and follow-up information was obtained from hospital records or directly from the patient's families. Patients were evaluated considering to age, tumor focality, tumor location, gleason score, pathological tumor stage, extracapsular extension, seminal vesicle invasion, surgical margin status, tumor volume and clinical significance. The serum prostate-specific

### antigen (PSA) levels were determined routinely before RCP

Histopathological findings were obtained surgically resected specimens from the Department of Pathology files that were evaluated by two pathologists. A routine pathological examination was used for all RCP specimens by sectioning and totally submitting the prostate. The prostate was severed from the bladder and then covered with India ink. After fixation for 24 h in 10% neutral buffered formalin, the prostate specimens were step sectioned at 3 mm intervals perpendicular to the long axis (apical-basal) of the gland. (Between 1997 and 2002, some of the prostate specimens were sliced at an interval of 5 mm). The apex, base and seminal vesicles were removed from each specimen and submitted in total for routine histological examination. The cut specimens examined histological as 2 μm-thick whole-mount haematoxylin and eosin (H&E)-stained sections. The stage of prostate cancer was based on the 2002 revision of the TNM system [7]. The Gleason score was determined using the 2005 International Society of Urological Pathology modified Gleason system [8]. Tumor volume was calculated using the length (L), width (W), and height (number of cross sections × sectional thickness, CST). According to Chen et al. derived the formula = 0.4 (slope of the regression line) × L × W × CST to estimate volume [9].

We defined clinically significant PCa features as any of the following: PCa tumor volume ≥ 0.5 cc, Gleason score ≥ 6, extraprostatic extension, seminal vesicle invasion, and/or a positive surgical margin according to the criterion advocated by Epstein et al. [10]. Data for the present study were identified by a structured MEDLINE search up to 1st September 2010. ''Bladder cancer'', ''cystoprostatectomy'', ''incidental'', and ''prostate cancer'' were the key words for searching the data. Only publications in English were considered. All the studies addressing the incidence, pathological characteristics, and/or clinical significance of prostate tumors were included and reviewed in detail

## Results

In this study, a total of 60 patients (20%) were incidentally diagnosed as having PCa in RCP specimens. Detailed characteristics of these 60 are summarized in Table 1. The mean age of the patients was 62 (range 51-75) years. All were acinar adenocarcinoma. Of the 20 adenocarcinoma (33.3%) were pT2a, whereas 12 (20%) and 22(36.6%) were pT2b and pT2c, respectively and 6 adenocarcinoma (10%) was T3a. Of these patients, 32 (53.3%) had clinically significant features. 12 (20%) had Gleason score of > 6, 37(61.7%) had PCa tumor volume ≥ 0.5 cc, 6(10%) had positive surgical margin and 6 (10%) had positive extraprostatic extension. Preoperative PSA was high in 5 patients (ranging between 10-29.05 ng/ml). The characteristics of their PCa were stage T2 in 2 of them and stage T3 in others. Tumor volume was significantly high in these patients. The surgical margin was positive in one of these patients. In 48 specimens (80%), the Gleason score was 6 or less. Negative margins were present in 54 (90%) of cases. Negative extraprostatic extension were present in 54 (90%) of cases. None of the patients had seminal vesicle invasion. All cases were pN0 for PCa. In 23 specimens (38.3%), the volume was less than 0.5 cc. Of the 60 cases of incidentally detected prostate cancer, 40 (66.7%) were considered clinically significant. Follow-up data were available for 60 patients. The cause of death was not related to the PCa in any of the patients and there was no PSA recurrence during follow-up of 96 months (range between 72-168 months).

**Table 1:** The clinicopathological characteristics of incidental prostate cancer at RCP in the 60 patients with bladder cancer

Features	Number of patient's n (%)
Mean age at surgery, years	62
Focality	
Monofocal	46
Multifocal	14
Tumor location	
Peripheral zone	40
Central zone	6
Transition zone	2
All three zones	12
Gleason score	
≤ 6	48
7 (3+4)	6
7 (4+3)	6
8-10	-
pT (TNM system)	
pT2a	20
pT2b	12
pT2c	22
pT3a	6
pT3b	-
pT4	-
Stage of bladder cancer	8
p T1	20
p T2a	26
p T2b	6
p T3a	
Seminal vesicle invasion	
Negative	60
Positive	-
Extraprostatic extension	
Negative	54
Positive	6
Surgical margin status	
Negative	54
Positive	6
Tumor volume, mL	
Range	23
< 0.5	37
≥ 0.5	
Clinically insignificant	20
Clinically significant	40

## Discussion

RCP represents the most effective treatment for muscle-invasive nonmetastatic bladder cancer [11]. Many authors have reported a higher prevalence of PCa in patients with bladder cancer [12,13], although data are sparse regarding the outcome of these patients [14].

The frequent high coincidence of prostate and bladder cancer occurring together could be explained by a common carcinogenic pathway. In this respect, Singh et al. reported that some tumor suppressor genes such as p53 and Rb play a major role in the development of both prostate and bladder cancers [15]. More recently, Amara et al. demonstrated that prostate stem cell antigen is overexpressed in most human transitional cell carcinomas in an immune-histochemical analysis [16]. However, these represent preliminary experiences and the model for a common carcinogenic pathway remains to be elucidated [1].

According to literature, the proportion of clinically significant cancers in the series published previously varies from 10% to 70% [4-6,12,14,17-33] [Table 2]. This high range between the proportions may be related with hereditary and exogenous factors, such as food consumption and patterns of sexual behavior. The detailed pathological examination of the excised prostatic tissue specimens may be another important factor for the detection of small cancer [31]. In this respect, two important issues are the thickness of the slice of the prostate and whether the prostate is totally embedded [3]. The advantage of complete sampling over partial sampling is that small foci of cancer are seen more frequently. When prostate slices are thicker than 5 mm, small foci of cancer might miss within the slice. By complete sampling, cancer features, such as extraprostatic extrusion and positive margins, are more accurately evaluated [16]. Regarding the published reports in Table 2, Mazzucchelli et al [3] found a 49.6% rate of incidental PCa, in RCP specimens with serial step slices taken at 2-3-mm intervals. However, the incidence was lower in studies using a different pathological examination protocol. For instance, Jin et al. identified PCa in 14% of the examined specimens when using 5-mm thick slices [31].

**Table 2:** PCa in cystoprostatectomy specimens: literature overview.

References	Year	No.of Patients	Mean age (year)	Slice Thickness (mm)	Sampling of prostate	No. of prostate cancer (%)	No. of significant prostate cancer (%)
Abbas [17]	1996	40	64.3	2-3	Partial	18(45)	6(33)
Moutzouris [18]	1999	59	66.5	5	Complete	16 (27)	NA
Aydin [19]	1999	121	67.1	NA	NA	17 (14.0)	NA
Yang [20]	1999	49	67	8 3	Complete	16 (33)	NA
Conrad [21]	2001	133	60	3	Complete	58 (43.6)	11 (19)
Prange [22]	2001	85	64	4	Complete	41(48)	4(10)
Cindolo [23]	2001	165	69	3	Partial/complete	17(10.3)	NA
Ward [24]	2004	129	69	NA	NA	30(23.3)	18(60)
Revelo [25]	2004	121	67.4	5	Complete	50(41.3)	24(48)
Kouriefs [26]	2005	128	NA	NA	NA	23(18.0)	NA
Delongchamps[14]	2005	141	62	4	Complete	20(14.2)	14(70)
Ruffion [12]	2005	100	62	2.5	Complete	51 (51.0)	6 (12)
Lee [6]	2006	248	63.5	NA	Complete	10 (4.0)	NA
Rocco [27]	2006	63	67	3	Complete	34 (54)	12 (35)
Abdelhady [5]	2007	204	67	NA	Complete	58 (28.4)	18 (31)
Winkler [32]	2007	97	NA	2	Partial	58 (60)	31 (53)
Hosseini [28]	2007	50	62.5	NA	Partial	7 (14)	4 (57)
Weizer [29]	2007	35	65	NA	NA	16 (46)	4 (25)
Jin [31]	2008	264	70.9	5	Complete	37 (14)	12 (32.4)
Mazzucchelli [4]	2009	248	68	3	Complete	123 (49.6)	23 (18.7)
Nakagawa [33]	2009	349	65	5	NA	91 (26.1)	68 (74.7)
Gakis [30]	2010	95	68	4-5	NA	26 (27)	7 (27)
*Present study *	*2010*	*300*	*62*	*3-5*	*Complete*	*60(20)*	*40(66.6)*

In our study between 1997 and 2002 the prostate specimens were sliced at an interval of 5 mm. At the same interval 160 patients were underwent RCP and only 14 patients (8.75%) with incidental PCa were identified. Probably several incidental cancers might have been missed in this interval. After 2002 prostate specimens were evaluated with slices taken every 3 mm from the base to the apex of the gland, as is usually done for RCP, there was incidental PCa in 46 of 140 patients (32.8%) who underwent RCP. We believe that using slices taken every 2-3 mm, could detect a higher incidence of PCa.

Stamey et al. first defined the clinically significant adenocarcinoma of PCa in RCP specimens [34]. According to these autours clinically significant prostate cancer is based on Gleason score, tumour volume and stage, lymph node status and resection margin [34]. After than Epstein et al. defined clinically insignificant PCa features: (1) a Gleason score ≤ 6 without Gleason pattern 4 or 5, (2) organ-confined disease (no extraprostatic extension, seminal vesicle invasion, or lymph node involvement and (3) a tumour volume < 0.5 cm3 [10]. We evaluated clinically significant PCa features as any of the following: PCa tumor volume > 0.5 cc, Gleason score > 6, extracapsular extension, seminal vesicle invasion, and/or a positive surgical margin according to the criterion advocated by Epstein et al. According to this definition, the ratio of clinically significant PCa in our study was 66.7% (40/60) and this is a remarkable ratio.

Careful preoperative evaluation to diagnose concurrent PCa is very important [30]. Some authors have tried to use PSA into predictive models of tumor significance. Stamey et al. reported that serum PSA had been associated with cancer volume [34]. Winkler investigated the relationship between PSA level and tumour volume for incidental adenocarcinoma of the prostate found in RCP specimens. According to this study, the median PSA level for patients with and without prostate cancer was not significantly different [32]. The correlation between tumor volume and PSA level is also controversial [32]. Although in our series, tumor volume was high in patients with elevated levels of PSA, the number of these patients is not enough to advocate this relationship. In patients with PSA > 4 ng/mL or a palpable nodule a meticulous dissection of prostate during RCP should be performed.

According to Delongchamps et al. the outcome of patients with incidental prostate ca after RCP depends on the prognosis of bladder tumor [14]. In their report on 141 patients, twenty of them had incidental PCa and no patient experienced PSA recurrence during the follow-up. [14]. Pritchett et al. reported no worse survival in patients with both cancers compared with those with bladder cancer alone [35]. Wolters et al demonstrated that screen detected prostate cancer treated with radical prostatectomy shows more aggressive features than incidentally found prostate cancer [36]. In our study, PSA did not reach the nadir < 0.2 ng/mL (considered the cut-off value of biochemical recurrence for PCa) in a mean follow-up of 96 months (range, 72-168 months). All of the patients with incidental PCa were still alive. These findings show that incidental PCa does not influence prognosis and suggest that the outcome of patients with incidentally discovered PCa after RCP depends on the prognosis of the bladder cancer.

## Conclusions

The reported incidence of PCa in RCP specimens is highly variable and mostly depending on the histopathology technique of sampling. PSA cannot identify asymptomatic PCa, so there is still no effective tool for the detection of PCa before surgery. In line with published reports, incidental PCa does not impact the prognosis of bladder cancer patients undergoing RCP. The clinical significance of these incidentally discovered cancers remains questionable, as the outcome of patients depends on the prognosis of the bladder tumor.

## Competing interests

The authors declare that they have no competing interests.

## Authors' contributions

BA and HV both participated equally in literature search, conceptualization and preparation of the manuscript. Both authors have read the manuscript and approve it for publication.

## References

[B1] Barbisan F Mazzucchelli R Scarpelli M Lopez-Beltran A Cheng L Kirkali Z Montironi R Urothelial and incidental prostate carcinoma in prostates from cystoprostatectomies for bladder cancer: is there a relationship between urothelial and prostate cancer? BJU Int 2009 103 1058 63 10.1111/j.1464-410X.2008.08207.x 19076141

[B2] Damiano R Di Lorenzo G Cantiello F De Sio M Perdonà S D'Armiento M Autorino R Clinicopathologic features of prostate adenocarcinoma incidentally discovered at the time of radical cystectomy: an evidence-based analysis Eur Urol 2007 52 648 57 10.1016/j.eururo.2007.06.016 17600614

[B3] Mazzucchelli R Barbisan F Scarpelli M Lopez-Beltran A van der Kwast TH Cheng L Montironi R Is incidentally detected prostate cancer in patients undergoing radical cystoprostatectomy clinically significant? Am J Clin Pathol 2009 131 279 83 10.1309/AJCP4OCYZBAN9TJU 19141388

[B4] Mazzucchelli R Barbisan F Santinelli A Scarpelli M Galosi AB Lopez-Beltran A Cheng L Kirkali Z Montironi R Prediction of prostatic involvement by urothelial carcinoma in radical cystoprostatectomy for bladder cancer Urology 2009 74 385 90 10.1016/j.urology.2009.03.010 19501882

[B5] Abdelhady M Abusamra A Pautler SE Chin JL Izawa JI Clinically significant prostate cancer found incidentally in radical cystoprostatectomy specimens BJU Int 2007 99 326 329 10.1111/j.1464-410X.2006.06558.x 17026595

[B6] Lee SH Chang PL Chen SM Sun GH Chen CL Shen BY Wu YS Tsui KH Synchronous primary carcinomas of the bladder and prostate Asian J Androl 2006 8 357 9 10.1111/j.1745-7262.2006.00129.x 16625287

[B7] Sobin LH Wittekind C Sobin LH, Wittekind C Urologic tumours: prostate TNM Classification of Malignant Tumours 2002 6 New York: Wiley and Liss 184 187

[B8] Montironi R Cheng L Lopez-Beltran A Scarpelli M Mazzucchelli R Mikuz G Kirkali Z Montorsi F Original Gleason system versus 2005 ISUP modified Gleason system: the importance of indicating which system is used in the patient's pathology and clinical reports Eur Urol 2010 58 369 73 10.1016/j.eururo.2010.04.028 20478652

[B9] Chen ME Johnston D Reyes AO Soto CP Babaian RJ Troncoso P A streamlined three-dimensional volume estimation method accurately classifies prostate tumors by volume Am J Surg Pathol 2003 27 1291 301 10.1097/00000478-200310000-00001 14508390

[B10] Epstein JI Walsh PC Carmichael M Brendler CB Pathologic and clinical findings to predict tumor extent of nonpalpable (stage T1c) prostate cancer JAMA 1994 271 368 74 10.1001/jama.271.5.368 7506797

[B11] Stein JP Skinner DG Radical cystectomy for invasive bladder cancer: long-term results of a standard procedure World J Urol 2006 24 296 304 10.1007/s00345-006-0061-7 16518661

[B12] Ruffion A Manel A Massoud W Decaussin M Berger N Paparel P Morel-Journel N Lopez JG Champetier D Devonec M Perrin P Preservation of prostate during radical cystectomy: evaluation of prevalence of prostate cancer associated with bladder cancer Urology 2005 65 703 7 10.1016/j.urology.2004.10.076 15833512

[B13] Autorino R Di Lorenzo G Damiano R Giannarini G De Sio M Cheng L Montironi R Pathology of the prostate in radical cystectomy specimens: a critical review Surg Oncol 2009 18 73 84 10.1016/j.suronc.2008.07.006 18760917

[B14] Delongchamps NB Mao K Theng H Zerbib M Debré B Peyromaure M Outcome of patients with fortuitous prostate cancer after radical cystoprostatectomy for bladder cancer Eur Urol 2005 48 946 50 10.1016/j.eururo.2005.07.008 16126325

[B15] Singh A Jones RF Friedman H Hathir S Soos G Zabo A Haas GP Expression of p53 and pRb in bladder and prostate cancers of patients having both cancers Anticancer Res 1999 19 5415 7 10697570

[B16] Amara N Palapattu GS Schrage M Gu Z Thomas GV Dorey F Said J Reiter RE Prostate stem cell antigen is overexpressed on human transitional cell carcinoma Cancer Res 2001 61 46605 11406532

[B17] Abbas F Hochberg D Civantos F Soloway M Incidental prostatic adenocarcinoma in patients undergoing cystoprostatectomy for bladder cancer Eur Urol 1996 30 322 6 8931964 10.1159/000474190

[B18] Moutzouris G Barbatis C Plastiras D Mertziotis N Katsifotis C Presvelos V Theodorou C Incidence and histological findings of unsuspected prostatic adenocarcinoma in radical cystoprostatectomy for transitional cell carcinoma of the bladder Scand J Urol Nephrol 1999 33 27 30 10100360

[B19] Aydin O Coşar EF Varinli S Buğdayci R Tansuğ Z Prostatic intraepithelial neoplasia in prostate specimens: frequency, significance and relationship to the sampling of the specimen (a retrospective study of 121 cases) Int Urol Nephrol 1999 31 687 697 10.1023/A:1007120907921 10755361

[B20] Yang CR Ou YC Ho HC Kao YL Cheng CL Chen JT Chen LP Ho WL Unsuspected prostate carcinoma and prostatic intraepithelial neoplasm in Taiwanese patients undergoing cystoprostatectomy Mol Urol 1999 3 33 39 10851294

[B21] Conrad S Hautmann SH Henke RP Erbersdobler A Simon J Straub M Graefen M Hautmann RE Huland H Detection and characterization of early prostate cancer by six systematic biopsies and fine needle aspiration cytology in prostates from bladder cancer patients Eur Urol 2001 39 25 29 10.1159/000052579 11340283

[B22] Prange W Erbersdobler A Hammerer P Graefen M Hautmann SH Hautmann RE Huland H Henke RP High-grade prostatic intraepithelial neoplasia in cystoprostatectomy specimens Eur Urol 2001 39 30 31 11340284 10.1159/000052580

[B23] Cindolo L Benincasa G Autorino R Domizio S De Rosa G Testa G D'Armiento M Altieri V Prevalence of silent prostatic adenocarcinoma in 165 patients undergone cystoprostatectomy: a retrospective study Oncol Rep 2001 8 269 271 11182038

[B24] Ward JF Bartsch G Sebo TJ Pinggera GM Blute ML Zincke H Pathologic characterization of prostate cancers with a very low serum prostate specific antigen (0-2 ng/mL) incidental to cystoprostatectomy: is PSA a useful indicator of clinical significance? Urol Oncol 2004 22 40 47 10.1016/S1078-1439(03)00093-0 14969803

[B25] Revelo MP Cookson MS Chang SS Shook MF Smith JA Jr Shappell SB Incidence and location of prostate and urothelial carcinoma in prostates from cystoprostatectomies: implications for possible apical sparing surgery J Urol 2004 171 646 651 10.1097/01.ju.0000107380.40481.bc 14713778

[B26] Kouriefs C Fazili T Masood S Naseem MS Mufti GR Incidentally detected prostate cancer in cystoprostatectomy specimens Urol Int 2005 75 213 216 10.1159/000087796 16215307

[B27] Rocco B de Cobelli O Leon ME Ferruti M Mastropasqua MG Matei DV Gazzano G Verweij F Scardino E Musi G Djavan B Rocco F Sensitivity and detection rate of a 12-core trans-perineal prostate biopsy: preliminary report Eur Urol 2006 49 827 833 10.1016/j.eururo.2005.12.021 16426731

[B28] Hosseini SY Danesh AK Parvin M Basiri A Javadzadeh T Safarinejad MR Nahabedian A Incidental prostatic adenocarcinoma in patients with PSA less than 4 ng/mL undergoing radical cystoprostatectomy for bladder cancer in Iranian men Int Braz J Urol 2007 33 167 173 10.1590/S1677-55382007000200006 17488535

[B29] Weizer AZ Shah RB Lee CT Gilbert SM Daignault S Montie JE Wood DP Jr Evaluation of the prostate peripheral zone/capsule in patients undergoing radical cystoprostatectomy: defining risk with prostate capsule sparing cystectomy Urol Oncol 2007 25 460 464 10.1016/j.urolonc.2006.09.016 18047952

[B30] Gakis G Schilling D Bedke J Sievert KD Stenzl A Incidental prostate cancer at radical cystoprostatectomy: implications for apex-sparing surgery BJU Int 2010 105 468 71 10.1111/j.1464-410X.2009.08739.x 20102366

[B31] Jin XD Chen ZD Wang B Cai SL Yao XL Jin BY Incidental prostate cancer in radical cystoprostatectomy specimens Asian J Androl 2008 10 809 14 10.1111/j.1745-7262.2008.00420.x 18645685

[B32] Winkler MH Livni N Mannion Hrouda D Christmas T Characteristics of incidental prostatic adenocarcinoma in contemporary radical cystoprostatectomy specimens BJU Int 2007 99 554 8 10.1111/j.1464-410X.2006.06660.x 17407514

[B33] Nakagawa T Kanai Y Komiyama M Fujimoto H Kakizoe T Characteristics of prostate cancers found in specimens removed by radical cystoprostatectomy for bladder cancer and their relationship with serum prostate-specific antigen level Cancer Sci 2009 100 1880 4 10.1111/j.1349-7006.2009.01267.x 19659608 PMC11158997

[B34] Stamey TA Freiha FS McNeal JE Redwine EA Whittemore AS Schmid HP Localized prostate cancer Relationship of tumor volume to clinical significance for treatment of prostate cancer. Cancer 1993 71 933 8 10.1002/1097-0142(19930201)71:3+<933::aid-cncr2820711408>3.0.co;2-l 7679045

[B35] Pritchett TR Moreno J Warner NE Lieskovsky G Nichols PW Cook BA Skinner DG Unsuspected prostatic adenocarcinoma in patients who have undergone radical cystoprostatectomy for transitional cell carcinoma of the bladder J Urol 1988 139 1214 6 3373591 10.1016/s0022-5347(17)42864-3

[B36] Wolters T Montironi R Mazzucchelli R Scarpelli M Roobol MJ van den Bergh RC van Leeuwen PJ Hoedemaeker RF van Leenders GJ Schröder FH van der Kwast TH Comparison of incidentally detected prostate cancer with screen-detected prostate cancer treated by prostatectomy Prostate 2011 10.1002/pros.21415 21538424

